# Critical Review of Comparative Study of Selective Laser Melting and Investment Casting for Thin-Walled Parts

**DOI:** 10.3390/ma16237346

**Published:** 2023-11-25

**Authors:** Naol Dessalegn Dejene, Hirpa G. Lemu, Endalkachew Mosisa Gutema

**Affiliations:** 1 Department of Mechanical and Structural Engineering and Materials Science, Faculty of Science and Technology, University of Stavanger, N-4036 Stavanger, Norway; 2 Department of Mechanical Engineering, College of Engineering &Technology, Wallaga University, Nekemte P.O. Box 395, Ethiopia; endalkem5@gmail.com

**Keywords:** additive manufacturing, casting, investment casting, selective laser melting, thin-walled, microstructure, porosity, residual stress, roughness, dimensional accuracy

## Abstract

Thin-walled structures are a significant and growing portion of engineering construction, with a wide range of applications, including storage vessels, industrial buildings, warehouses, aircraft, automobiles, bridges, ships, and oil rigs. Thin-walled components with minimum thickness without compromising strength and other quality characteristics are the desire of modern industry. Reducing wall thickness not only aids in lowering the cost of production. It also improves the effectiveness of engineering systems, resulting in lower fuel consumption and lower emissions of hazardous gases to the environment. Nowadays, even though thin-walled parts are demanded, the constraints of the production process, quality, and reliability are the concerns of current research and development. The ability to produce parts with intricate geometries and tight dimensional tolerances are important criteria for advanced manufacturing processes. In the early days of society, investment casting was used to produce jewelry, weapons, and statues. In modern industry, investment casting is still used to produce thin-walled and intricate parts such as turbine blades. The current advancements in SLM, which has the capacity to produce thin-walled and intricate parts, have recently attracted attention due to several benefits, such as the supreme degree of design freedom and the viability of tool-free production directly from CAD data. However, the current technological applications of SLM and investment casting are crucial for producing parts at the desired quality and reliability. This review article focuses on comparative studies of SLM and investment casting at the current technology level. The basis of comparison via systematic approach is mechanical characterization; quality in terms of porosity, microstructure, surface roughness and dimensional accuracy; and residual stress. Therefore, the latest open scientific sources published are considered to obtain sufficient literature coverage. Better tensile strength and fine microstructure are found in SLM, while better surface quality, fatigue load resistance, ductility, and residual stress are found in investment casting. The research gap for further investigation is indicated.

## 1. Introduction

The imperative to reduce the weight of cast components in jet engines has become increasingly crucial due to stringent global regulations aimed at mitigating fuel consumption and emissions. This imperative extends beyond the aerospace industry, permeating various sectors. The capacity to manufacture components with thinner walls not only translates to a reduction in production costs and resource consumption but also signifies a marked enhancement in the efficiency of engineering systems. This, in turn, leads to an evident decrease in fuel consumption and a subsequent reduction in the emission of hazardous gases into the environment. In essence, the drive toward lighter cast components represents a pivotal step toward more sustainable and eco-conscious industrial practices and transportation in the future.

Nowadays, thin-walled components are widely used in several parts of the aerospace, power engineering, electronic equipment, and biomedical industries [1,2], where light weight, high accuracy, and ergonomics are demanded. Moreover, thin-walled structures are a significant and growing portion of engineering construction, with a wide range of applications, including storage vessels, industrial buildings, warehouses, aircraft, automobiles, bridges, ships, and oil rigs [2]. The thin wall thickness is needed for lighter weight, less material consumption, and less wastage without compromising the desired strength. For instance, in aerospace industries thin-walled parts are required to minimize fuel consumption and resource utilization [3], which reduces operational costs; moreover, greater fuel consumption pollutes the environment. The development of additive manufacturing (AM), commonly known as 3D printing, has been conducted in a transformative era in production methods. Unlike traditional manufacturing processes that involve subtracting material from a solid block, AM techniques build objects layer by layer from digital designs. This revolutionary approach has the potential to revolutionize industries by offering greater design freedom, reduced production times, and increased efficiency. Importantly, AM also holds promise in addressing environmental concerns associated with traditional manufacturing [4]. By enabling more precise material usage, AM can significantly reduce waste, leading to a decrease in the negative environmental effects typically associated with excessive resource consumption. Furthermore, AM allows for the use of recycled or sustainable materials, further contributing to eco-friendliness. As technology continues to advance, it holds the promise of not only meeting functional product requirements but also making significant strides toward a more sustainable and environmentally responsible manufacturing future. 

The AM techniques in the current sector are advancing from day to day [5,6,7]. According to Lemu [8], the concept of AM holds a pivotal role in what is commonly referred to as “Industry 4.0”. This term encompasses the ongoing transformation of industrial processes, characterized by a convergence of cutting-edge technologies. At its core, AM represents a fundamental shift in manufacturing methodology. It allows for the creation of complex and customized objects by layering materials, as opposed to traditional subtractive manufacturing methods. In the context of Industry 4.0, AM is not an isolated technology but rather a key enabler. It works hand in hand with automation, the Internet of Things (IoT), big data analytics, and the digitization of manufacturing processes. Together, these components form a sophisticated ecosystem where machines communicate, adapt, and optimize production autonomously. AM’s ability to promptly prototype, produce on demand, and even customize products aligns perfectly with the agile and data-driven nature of this new industrial era. It has the potential to revolutionize supply chains, reduce waste, and accelerate innovation across various industries, making it an indisputable cornerstone of Industry 4.0. The application of AM in high-tech industries is increasing and its products becoming more commercialized [9]. Companies must apply cutting-edge technologies and develop a set of measures that gauge the advantages of the shift to align with a green product lifecycle management vision. According to Raja et al. [10], most industries have begun employing 3D printed components, which are quickly replacing traditional material components. The need to reduce the weight of cast components in jet engines is growing as a result of global regulations that aim to reduce fuel consumption and emissions [3]. 

In today’s manufacturing landscape, there is a growing demand for thin-walled parts, driven by various industries seeking lightweight and efficient components [11,12]. However, this demand comes with its set of challenges and concerns, which are at the forefront of current research and development efforts. These challenges encompass the intricacies of the production process, such as the need for specialized techniques to create thin-walled structures, ensuring precision and efficiency while avoiding defects. Moreover, maintaining the quality and reliability of these parts remains a paramount concern, as thinner walls can make components more susceptible to structural weaknesses and performance issues. Thus, ongoing research and development endeavors are focused on addressing these constraints to meet the evolving requirements of modern manufacturing. 

The ability to produce parts with intricate geometries and tight dimensional tolerances are important criteria for advanced manufacturing processes. In the early days of society, investment casting was used to produce jewelry, weapons [3,7], and statues [13]. Some of the applications of investment casting in modern industries are: turbine blades [1,14], jewelry castings, airplane parts, modern weapons [14], and other industrial/scientific components [1]. Manufacturing techniques have been advancing progressively in the last three decades since the emergence of AM technology in the late 1980s [15,16]. AM is a rapid bottom-to-top free-form solid manufacturing technology that produces parts with intricate designs by gradually adding materials to build three-dimensional objects. This technique can significantly decrease material waste and accelerate the development of new products to produce customized parts [17]. 

SLM has recently attracted more attention because of its many benefits, such as the supreme degree of design freedom and the viability of tool-free production directly from CAD data. SLM and investment casting are two manufacturing processes that share common application areas, such as the aerospace and automotive industries, where complex and high-performance components are required. Both methods offer precise control over geometry and can produce intricate shapes. However, they differ in their approach; SLM is an AM process that fuses layers of metal powder with a laser, allowing for rapid prototyping and customization, but it may have limitations in terms of size and post-processing. In contrast, investment casting is a technique that involves creating a mold and pouring molten metal into it, offering versatility in materials and scalability but requiring more time and cost for tooling [18,19,20]. Due to the rapid advancement of technology, manufacturing techniques have increased in prominence, and industries are prioritizing the discovery of quicker approaches [21]. The choice between these techniques depends on specific project requirements, including material properties, production volume, and lead time.

Investment casting stands as the prevailing production method in many industries, primarily because it addresses the inherent challenges associated with the weldability, formability, and machinability of certain alloys like cobalt-chrome, which are commonly employed for complex component manufacturing [22]. However, the advent of SLM has been accompanied by fresh processing possibilities, prompting a crucial comparison between the two techniques to assess their respective impacts on product quality.

Previous scholars have undertaken efforts to study both techniques, with a particular focus on using investment casting as a benchmark for adapting SLM processes. These studies have aimed to assess the viability and efficiency of incorporating SLM technology into traditional investment casting methods, potentially offering improvements in terms of precision, material utilization, and production speed. By leveraging the knowledge and standards established by investment casting, researchers seek to refine and optimize SLM adaptations, ultimately advancing the field of additive manufacturing while maintaining a link to established manufacturing practices. The claim is that any metal suitable for traditional casting or welding techniques can also be utilized in SLM additive manufacturing [23,24,25]. This implies that SLM technology offers versatility in material selection, potentially expanding its applicability across various industries. However, the success of using a specific metal in SLM may still depend on its specific properties and compatibility with the process. This review article aims to establish a common understanding between two prominent manufacturing techniques, selective laser melting and investment casting, by conducting a comprehensive comparative study within the context of current technological advancements. The comparison is rooted in a systematic approach that encompasses various critical aspects, including mechanical characterization and quality parameters such as porosity, microstructure, surface roughness, and dimensional accuracy, as well as an assessment of residual stress. To ensure the most up-to-date and relevant information, this study primarily relies on the latest open scientific sources published in the last decade. By inspecting and analyzing the findings of these sources, this review seeks to shed light on the strengths, weaknesses, and overall performance of SLM and investment casting in the contemporary manufacturing landscape, providing valuable insights for researchers, engineers, and industry professionals in the field of advanced manufacturing and materials science.

## 2. Investment Casting for Thin-Walled Parts

**Figure 1:**
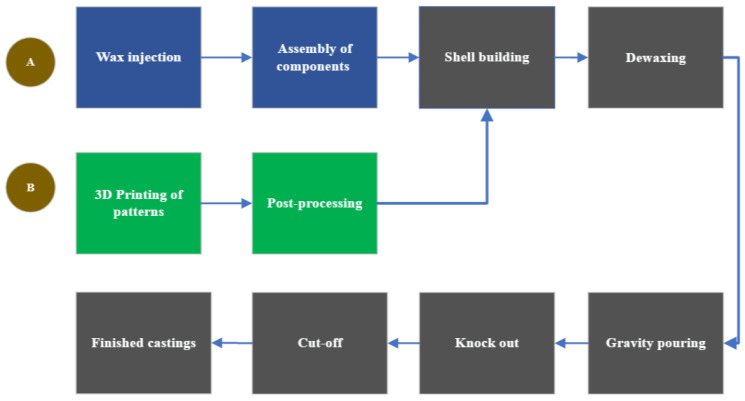
Investment casting process (Path A) and rapid investment casting (Path B).

Investment casting, often known as lost-wax or precision casting, stands as one of the earliest manufacturing techniques, with roots tracing back thousands of years [26]. This fascinating and adaptable technique involves crafting intricate metal components through a multi-step process: first carefully producing a wax model of the desired part, then encasing it in a ceramic shell before finally melting away the wax, leaving a hollow mold. The flow is illustrated in Figure 1, tracing the flow of investment casting along path A. Molten metal is then poured into this cavity, assuming the precise shape of the original wax model as it solidifies. Investment casting excels in crafting complex, highly detailed, and dimensionally accurate components, making it a cornerstone in the production of aerospace components, jewelry, and critical engineering parts where precision and fine detailing are paramount. Egyptians utilized this method during the reign of the pharaohs to create jewelry out of gold, copper, and bronze [27]. Even though there is no clear evidence of its origin, it is certainly mentioned as the oldest manufacture in metallurgy. The investment casting technique made significant contributions to ancient civilizations. Furthermore, when traditional tooling processes failed to meet the increased demand for war equipment during the Second World War in the United States, investment casting was used to produce turbine blades, aircraft engines, and other items [14,28]. 

Investment casting is known by different names, such as lost wax casting [4] or precision casting [1]. Investment casting excels in creating parts with superior surface quality, precise dimensions, and intricate shapes. It involves creating a wax pattern, coating it in ceramic, melting away the wax, and pouring molten metal into the ceramic mold. This process allows for intricate details and minimal post-processing, making it ideal for aerospace, automotive, and jewelry industries. Its versatility and precision make investment casting a preferred choice for producing high-quality components [4,14,29]. According to Rani et al. [30], investment casting produces near-net parts, which do not require the involvement of machining, so that it is known as precision casting. The lost wax process name is acquired from the wax substance frequently used to make patterns. A wax pattern that mimics the finished cast part is made during this process, typically using a ceramic mold. The wax pattern is then covered with refractory ceramic material and dried. Next, the wax pattern with ceramic material assembly is heated to remove the wax, and a mold made from the refractory material is produced [31,32].

As mentioned earlier, lightweight and thin-walled structures are in high demand in high-tech industries. For example, gas turbine blades, which work in challenging environmental conditions, require a high degree of dimensional accuracy as well as being thin-walled [29]. The turbine industry is now more regularly demanding the production of intricate, thin-walled cast components [3]. One way to reduce the weight of an engineering system is to use lightweight materials or integrated multifunctional components. Investment casting has the capacity to produce thin-walled components, i.e., reduce the overall weight of systems [3]. According to Naplocha et al. [33], among various manufacturing techniques, investment casting allows for the fabrication of thin-walled complex shapes with outstanding surfaces and dimensional accuracy. However, the challenges of producing thin-walled parts by investment casting are often inadequate filling, shrinkage, porosity, and cold shuts due to premature solidification and long-distance feeding [34]. Moreover, the drawbacks of investment casting are that it is a labor intensive and time-consuming as well as an expensive process due to high tooling costs for producing wax patterns [1,35]. Investment casting is used to produce weapons, jewelry, and other complex parts. These days, it is used for a variety of industrial and scientific research parts, such as turbine blades and other fine items [1]. Currently, the most competent manufacturing techniques are coming to the fore to implement investment casting, with various advantages and drawbacks due to the current level of the technology. The current article reviews numerous studies conducted by researchers at various stages of investment casting and SLM development to emphasize their significance and indicate examples of the technology.

Investment casting has emerged as the foremost manufacturing technique, showcasing remarkable competence in various industries. This method, characterized by its ability to produce intricate and highly detailed parts, offers a unique set of advantages and drawbacks within the current technological landscape. The precision and intricacy achievable through investment casting make it a preferred choice for applications where fine detail and complexity are paramount. However, it is not without its limitations, such as higher production costs and longer lead times compared to other methods. The current article critically examines a multitude of studies conducted by researchers across different stages of investment casting and selective laser melting, underscoring their pivotal contributions to the field. These studies shed light on the evolving significance of investment casting technology, emphasizing its potential to revolutionize manufacturing processes. By highlighting the advancements and challenges in this domain, the article aims to provide a comprehensive overview of the current state of the art in investment casting, ultimately shaping the trajectory of this transformative technology.

## 3. Selective Laser Melting for Thin-Walled Parts

**Figure 2:**
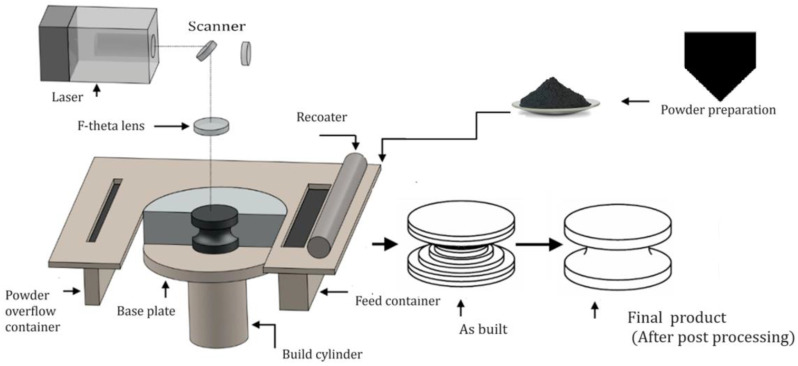
Selective laser melting process schematic diagram. (Adapted from [55] with permission, license number: 5643130085972).

The procedure referred to as rapid prototyping goes by numerous names, including layer production, additive manufacturing (AM), additive fabrication, additive layer manufacturing, rapid manufacturing, freeform fabrication, tool-free manufacturing, and direct digital manufacturing [36]. According to Vaudreuil et al. [37], AM constitutes a significant innovation in the automotive, biomedical, and aerospace industries. Its versatility and precision have made it indispensable in various fields, such as aerospace, where it is used to produce complex, lightweight components that enhance fuel efficiency and reduce emissions [38]. PBF technology, classified as one of the seven categories of AM in accordance with ISO/ASTM 529000:2015 [39], plays a pivotal role in a multitude of industry sectors [1]. In the energy sector, PBF is utilized to produce intricate parts for turbines and power plants, optimizing energy generation and distribution. Transportation benefits from PBF’s ability to manufacture custom components, enhancing vehicle performance and safety [40]. In the biomedical realm, it enables the production of patient-specific implants and prosthetics, revolutionizing healthcare [41,42]. Moreover, PBF technology has applications in the automotive industry, where it aids in crafting lightweight, durable parts, as well as in the jewelry industry for crafting intricate designs. The widespread utilization of PBF underscores its significance as a driving force behind technological innovation across these diverse sectors, propelling them into a future marked by efficiency, customization, and precision. The most advanced PBF method, known as laser powder bed fusion (LPBF) or SLM, enables the direct production of complex components from metal powder. As shown in Figure 2, metal powder is carefully layered on a platform to start. The powder is then melted layer by layer by a powerful laser and solidified, producing the final product. Post-processing could be required for surface finish and final qualities. SLM traces the geometry of each segment layer from a 3D model on the surface of the powder bed using thermal energy from a laser [43,44,45,46]. SLM has the capacity to produce thin-walled components [45,47]. Numerous research papers have explored the fabrication of thin-walled structures using SLM. These studies delve into SLM’s capabilities in producing intricate and lightweight components for various industries. Their findings contribute to advancing AM techniques and applications [48,49,50,51]. Studies have shown that the quality and reliability of thin-walled products produced using SLM are significantly influenced by various process parameters, including powder layer thickness, hatching distance, and laser power [50,52]. Adjusting these parameters is crucial for achieving desired results and ensuring the structural integrity of the manufactured components. Careful control and optimization of SLM process parameters are essential for producing high-quality thin-walled parts [53]. Moreover, Wu et al. [54] conducted an investigation on the limits of SLM’s ability to manufacture thin-walled components. Rapid cooling causes thermal shrinkage in the SLM process, which increases residual tension and makes it more difficult to fabricate thin walls. While the powder size, scan strategy, and part geometry regulate the dimensional accuracy, the parameters and machine settings establish a minimal wall thickness limit.

## 4. Rapid Investment Casting

Rapid investment casting (RIC) technology, driven by advancements in 3D printing techniques, represents a transformative evolution in the field of manufacturing and foundry processes [56,57]. The manual wax pattern preparation, including the assembly steps in the case of investment casting, is replaced by 3D printing in the RIC process, as shown in Figure 1, path B. Traditional investment casting is a labor-intensive and time-consuming process for making complex metal components. However, the integration of 3D printing technologies has revolutionized this age-old process, significantly reducing lead times and costs while enhancing design flexibility and precision [58]. Rapid development has become an essential tool for reducing the time it takes for a product to reach the market and as a productivity-boosting strategy [59].

One of the key benefits of rapid investment in casting technology is its ability to produce complex, high-quality metal parts with supreme accuracy [60,61]. Rapid investment casting uses 3D printing techniques to construct complex patterns or molds precisely and quickly, i.e., improved surface roughness and precision [62]. This results in better surface finishes and dimensional precision than traditional investment casting, which could introduce faults during the creation of wax or plastic patterns. This level of accuracy is particularly valuable in fields such as aerospace, healthcare, and the automotive industry, where there is a significant need for lightweight, high-performance parts. In a study by Kumar et al. [63], 316L stainless steel was cast, and the hardness, surface finish, and dimensional accuracy were tested. The results showed that the castings had good dimensional accuracy and a good surface finish. Additionally, the castings’ hardness levels were within the acceptable range for biomedical implants. The study’s conclusion proved the potential of using fused deposition modelling (FDM) to develop patterns for medical implants. It was promising technology for such applications, since it produced exact dimensions and satisfied the required hardness standards. In addition, according to Singh et al. [64], research successfully demonstrated a comprehensive approach to producing biomedical implants with desirable properties, encompassing surface quality, dimensional accuracy, hardness, and biocompatibility. This work opens promising avenues to producing high-quality implants for medical applications and tissue engineering.

RIC also provides impressive cost and time savings [65]. In comparison, 3D printing reduces lead times from weeks to days by doing away with the necessity for pattern generation and simplifying the manufacturing of molds. This effectiveness decreases production costs while simultaneously speeding up product development. More design freedom and customization are also made possible by quick investment in casting technology. Without the limits of conventional tooling, engineers and designers may quickly iterate on prototypes and make adjustments [66]. 

In general, RIC incorporates 3D printing techniques, and transforms the labor-intensive casting process by producing precise, high-quality metal parts quickly and relatively affordably. This technology, which provides design freedom and shortens lead times, is essential for sectors that demand intricate components. It significantly reduces costs and lead times while excelling in precision and outperforming conventional techniques. RIC encourages innovation since it offers design freedom without the limitations of conventional tooling, which makes it indispensable for complicated, specialized parts in a variety of industries.

RIC in the field of advanced manufacturing has both benefits and drawbacks. The surface roughness of RIC parts, which is usually rougher than that of components made using conventional investment casting techniques, is one of the significant challenges. To fulfil industrial standards, further post-processing operations, including polishing, may be required to achieve the appropriate surface quality. The range of materials suitable for this process is constrained in comparison to the wider spectrum available in traditional casting procedures, which is another issue with RIC. While RIC is shown to be a cost-effective method for intricate and complex products, it might not always be the best option for simpler or high-volume production because of the high costs of 3D printing materials and machinery. Moreover, building components for RIC needs careful consideration of the fundamental limitations resulting from 3D printing and investment casting, which may make some design elements difficult or impossible to implement. Furthermore, RIC parts commonly require post-processing, such as support and shell removal, which can increase production times and increase costs. While RIC is faster than conventional investment casting, there may be tradeoffs in terms of surface polish and material qualities; therefore, finding the careful balance between speed and quality continues to be a considerable problem.

## 5. Comparative Review of Components Produced by Selective Laser Melting and Investment Casting

SLM and investment casting are two distinct manufacturing methods with varying processes, yet they find common application in mission-oriented sectors such as aerospace, healthcare, and the automotive industry. Investment casting excels in producing intricate, high-precision parts through a process involving molds and molten metal. Conversely, SLM utilizes 3D printing technology to layer and fuse metal powders, enabling rapid prototyping and complex geometries. Both methods are valued in these industries for their ability to create components that meet stringent performance and quality standards, showcasing their versatility and importance in mission-critical applications [66]. These industries demand components that meet stringent standards of quality, reliability, and performance. However, choosing between SLM and investment casting involves considering numerous factors such as material complexity, production volume, and cost-effectiveness. Each method offers distinct advantages, making it a complex decision that depends on specific project requirements and constraints. The final choice should align with the desired outcome and resource allocation [67]. Both SLM and investment casting, despite their intrinsic disparities, find their niche in addressing the complex needs of these industries. SLM, as an additive manufacturing process, empowers engineers and designers to craft intricate and customized components by melting and fusing powdered material layer by layer using precision lasers [68].

The demand for complex, thin-walled parts in these sectors has surged, driven by imperatives such as weight reduction, enhanced performance, and miniaturization [65,66]. However, determining which manufacturing method is superior for achieving the desired level of quality, reliability, and cost-efficiency in producing such components remains a formidable challenge. To streamline this decision-making process, a comprehensive study delves into several key parameters. Mechanical characteristics, surface roughness, dimensional accuracy, microstructure, porosity, and residual stress of thin-walled parts produced through both SLM and investment casting have been accurately studied. The objective is to gain a holistic understanding of how these components perform under different manufacturing conditions and to ascertain which method is better suited for specific applications. This research effort underscores the importance of striking a delicate balance between quality, cost-effectiveness, and reliability. While SLM offers the advantages of intricate designs and customization, investment casting leverages well-established techniques for certain applications. The choice between these methods should be driven by evaluation of these critical parameters, aligning with the specific needs of the mission-oriented industry. 

In essence, this ongoing exploration of manufacturing techniques contributes significantly to the continual evolution of production processes in vital sectors. It ensures that the highest standards are not merely met but consistently exceeded, all while optimizing efficiency and cost-effectiveness. As these industries forge ahead for excellence, the driving force is to find the perfect manufacturing solution for each unique challenge.

### 5.1. Mechanical Properties

AM, particularly SLM, has garnered significant interest from mission-driven sectors such as aerospace, healthcare, and the automotive industry due to its precision, customization capabilities, and potential for lightweight and complex component production. Its ability to reduce material waste and enhance design flexibility makes it a compelling choice for advancing innovation in these critical industries [44,68]. However, in recent years research has focused on the mechanical performance of SLM-produced parts, their reliability for desired applications, and whether SLM can really replace investment casting. A study by Fotovvati et al. [69] investigated the deficiencies of SLM-produced parts in mechanical performance, chemical behavior, and thermal characteristics under dynamic and static interactions. Despite the high potential of SLM, having reliable mechanical properties in the produced components is essential for serious application production.

Superalloys are typically used in the production of parts for engineering systems, like jet engines and gas turbines, that operate at high temperatures. Titanium alloy Ti-6Al-4V is one of high interest to various aerospace industries. Leuders et al. [70] studied Ti-6Al-4V structural components produced by both SLM and investment casting under cyclic loading conditions, which are of critical interest to aerospace industries. The experimental investigation involved building lab-scale specimens at a constant layer thickness of 30 µm, 200 °C platform pre-heating temperature, and average particle size distribution (PSD) of 40 µm. Both types of specimens were built using the SLM technique in the z-direction, matching the construction direction to the loading axis in tensile and fatigue testing. Tests on lab-scale specimens and demonstrator components have verified that fatigue loadings are still difficult for Ti6Al4V produced via SLM compared to investment casting-produced parts. The surface roughness adversely affects the fatigue performance of SLM-produced parts [71]. The presence of rough surfaces and voids causes crack initiation, which leads to low fatigue load resistance [72]. The performance of parts produced through SLM under fatigue loads is a critical aspect that demands focused investigation. SLM, with its precision and versatility, stands as the leading candidate to supplant traditional casting methods within high-tech industries. Understanding and enhancing the fatigue resistance of SLM components will be pivotal in realizing its full potential and ensuring the reliability of critical applications. This area of research is essential for the continued advancement and adoption of SLM technology in various industrial sectors.

AlSi7Mg0.6 alloy is commonly used in the automotive and aerospace industries. This material has been widely used due to its excellent castability and corrosion resistance [69,70]. Al-Si alloys’ application in those industries roughly accounts for 80–90% of products [71,72]. SLM is a ground-breaking technology that encourages foundry companies to investigate its potential in these sectors. However, unlike with casting, there is less in-depth understanding of the mechanical properties and their relationship to microstructures in parts obtained using SLM. Pereira et al. [73] compared the AlSi7Mg0.6 alloy’s microstructure and mechanical characteristics as formed by the SLM and investment casting processes. The results obtained for the comparison of tensile strengths are shown in Table 1. In Pereira et al.’s research, a comparison between SLM and conventional as-cast components revealed that SLM samples demonstrated significantly higher tensile strength. This difference can be attributed to the unique microstructure and finer grain size achieved through the SLM process. The inherent precision of SLM enables a more uniform distribution of grains, enhancing mechanical properties. This finding underscores the potential of additive manufacturing techniques like SLM to produce superior mechanical performance in manufactured components. The work of Leuders et al. [70] on Ti-6Al-4V also showed that the ultimate tensile strength and the elongation of SLM-produced specimens was 12% greater than investment casting-produced samples. Moreover, the previous studies demonstrated that the SLM-fabricated parts had sufficient mechanical qualities for clinical usage [72]. For instance, the research findings indicate the material properties of Co-Cr produced by SLM has greater tensile strength and fracture toughness characteristics compared to investment casting [74,75]. Moreover, for medical applications, the corrosion resistance data for manufactured parts are crucial. Corrosion data, a great complement to cytotoxicity experiments in the evaluation of biocompatibility, demonstrates that SLM-fabricated samples had lower ion emission rates than cast ones [76]. They were also shown to be safe, non-irritating, and non-toxic to oral tissues and the body via cytotoxicity experiments [75]. Furthermore, according to the research conducted on Inconel718 for parts manufactured in both SLM and IC, the ductility measured in SLM samples through elongation at rupture is satisfactory for both horizonal (Y) and vertical (Z) orientations [33]. This exceeds the typical values required for aeronautical parts obtained by investment casting. After comparing the mechanical properties obtained in both processes, the strength of SLM samples in as-built and direct-aged conditions exceeded the strength obtained by investment-cast heat-treated material (TT0). The research conducted on gold jewelry applications clearly demonstrates that specimens produced using SLM technology have higher tensile strength but lower ductility than specimens produced using investment casting [77]. It seems that SLM is preferred in jewelry if the surface roughness is managed via controlling process parameters, including the powder PSDs. In another case, the study by Song et al. [78] indicates that SLM components displayed significant anisotropy related to building direction and poor ductility, but high yield tensile strength, UTS, and hardness. SLM-fabricated components exhibit static characteristics analogous to cast parts. Particularly, investment-cast parts showcase superior ductility, attributed to the anisotropic nature of SLM components. Nonetheless, advancements in SLM technology are imperative. Under precise process parameters, SLM parts overtake investment-cast parts in terms of tensile yield strength, ultimate tensile strength (UTS), and elongation percentage [71,73,74,75,79].

### 5.2. Surface Roughness and Dimensional Accuracy

Surface roughness and dimensional accuracy play a critical role in assessing the quality of manufactured parts. These factors are particularly crucial when dealing with thin-walled components, where even slight deviations can impact performance. Investment casting stands out as a manufacturing process renowned for its ability to achieve precise dimensional accuracy and produce parts with exceptionally smooth surface finishes. This accuracy and quality make investment casting a preferred choice for applications where precision and aesthetics are paramount, such as aerospace components or high-end jewelry [77,78,79,80], where the tolerance range normally achievable is ±1% of the nominal size, with a minimum of ±0.10 mm for dimensions lower than 10 mm and a minimum roughness of 3.2 µm [26]. Common dimensional errors often stem from dimensional shrinkage caused by fluctuations in process parameters. These variations can lead to inaccuracies in the final product’s size and dimensions. To mitigate such errors, maintaining consistent process conditions is crucial.

A significant drawback of the SLM process lies in the precision of the produced components, the ability to maintain control over their accuracy, and the consistency of the process regarding precision [81]. Surface roughness in investment casting and SLM-manufactured parts is a less-explored aspect, gathering limited attention in literature. Examining the surface finish condition is crucial for comprehensive comparison. Understanding how both processes affect surface quality can aid in making informed manufacturing choices, ensuring desired product outcomes. Exploring this side sheds light on the relative merits of these techniques. Castellanos et al. [82] conducted comparative study of roughness and micro hardness on impellers manufactured by both SLM and investment casting. Comparing surface finish measurements, SLM yielded an average surface roughness of 2.976 µm, accompanied by an arithmetic mean roughness of 3.388 µm. Conversely, investment casting demonstrated a slightly smoother surface, with average surface roughness measuring 2.819 µm and arithmetic mean roughness at 2.407 µm. According to Castellanos et al. [82], the tested geometric accuracy and surface roughness of turbine blades revealed investment casting values between −0.09 mm and +0.08 mm, exhibiting the least dimensional variance. The values for the SLM portions ranged from −0.12 to +0.13 mm.

Investment casting produces parts with smoother surfaces and more precise dimensions compared to parts produced through SLM. This means that when the conditions of the investment casting process are carefully controlled, they lead to better results in terms of surface roughness and accuracy in size and shape. These findings are supported by certain evidence presented in figures 1 and 2 of Ref. [82].

### 5.3. Microstructure and Porosity

The microstructure and porosity of SLM and investment casting are highly affected by the involvement of various process parameters. According to the study by Roudnicka et al. [23] on the microstructure, tensile strength, and hardness of Co-28Cr-6Mo, the microstructure of the cast result was relatively coarse due to the slow melt solidification at the casting temperature of around 1550 °C (tens of °C/min). Hence, it is composed of dendritic coarse (100–1000 µm) equiaxed grains. The SLM part produced shows fine dendritic structure [23,71] due to rapid solidification (about 104 °C/s). In the research conducted by Periera et al. [33], they focused on investigating the microstructure and mechanical characteristics of IN718 alloy through two manufacturing techniques, SLM and investment casting. Their findings revealed that SLM-produced components exhibited an exceptionally fine cellular microstructure. This fine microstructure was found to significantly enhance the overall strength of the manufactured parts. The study highlights the potential of SLM as a promising manufacturing method for achieving improved mechanical properties in materials like IN718 alloy.

**Figure 3:**
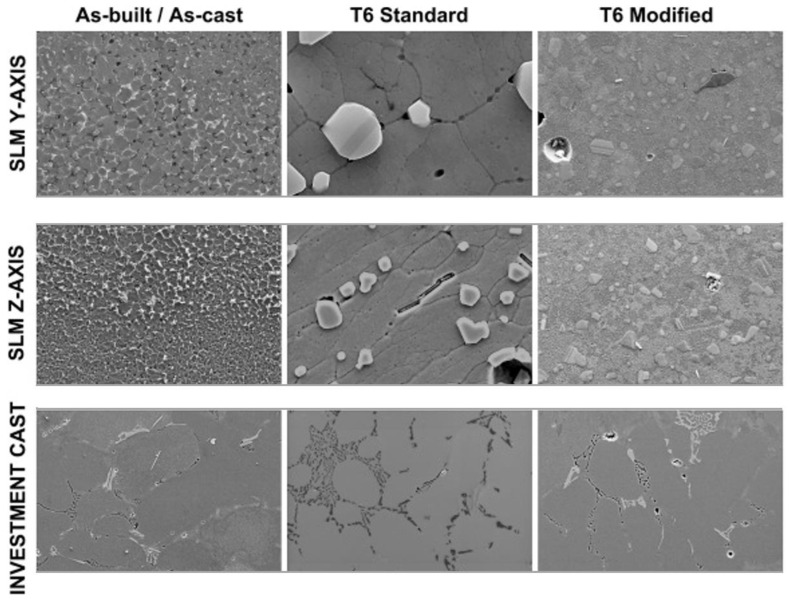
Field emission scanning electron micrographs for AlSi7Mg0.6 in as-built/as-cast and heat-treated conditions. SLM samples etched (HF 1%) and investment casting samples only polished. (Reused by permission from [73], license Number 5643131487779).

Another study on jewelry showed that the grain structure of specimens produced using SLM and investment casting differ significantly. SLM specimens have a grain size of about 10 microns, whereas investment casting specimens have a grain size of about 90 microns [77]. As shown in Figure 3, due to the rapid cooling rates involved in the SLM process, cellular microstructure occurs rather than the dendritic structure observed in investment casting samples. The other factor considered is that SLM uses fine powder particles, which leads to a more homogenous structure by forming a micro melt pool, leading to a high cooling rate (103 to 108 K/s) compared to the casting process [78]. 

Porosity stands out as a common flaw in SLM components, exerting a substantial influence on their functionality [44,83,84]. Comprehensive assessment is imperative, encompassing both the total porosity and the intricate structure and arrangement of individual pores. An understanding of the distribution of different defect types, such as fusing flaws and keyhole pores, is crucial. It is vital to examine how process variables like laser power and scan speed impact the spreading of these defects. This knowledge enables optimization of SLM processes, ensuring the production of high-quality components with minimal porosity-related performance issues [85]. Porosity is undesirable as it increases the susceptibility to corrosion, such as crevice corrosion and pitting corrosion [86]. Theoretically, the SLM method can produce structures with up to 100% nominal densities of the sintered alloy [85], but this is highly reliant on setting the operating conditions, such as the laser power, scan spacing, scan rate, and scan thickness, correctly [87]. Porosity is a well-known drawback of cast structures, which is associated with casting shrinkage and is still of a serious problem [88]. Shrinkage porosity is categorized as micro or macro [89]. This flaw is highly dependent on the design of the gating system, casting geometry, and running process factors [90]. If casting parameters are not properly controlled, volumetric shrinkage is formed, which tends to produce shrinkage porosity [91]. The formation of porosity in both SLM and investment casting is a very common and serious challenge. However, the comparative studies of those manufacturing techniques based on their level porosity formation and the factors needed to control it do not receive coverage.

### 5.4. Residual Stress in SLM and Investment Casting-Produced Thin-Walled Parts

**Figure 4:**
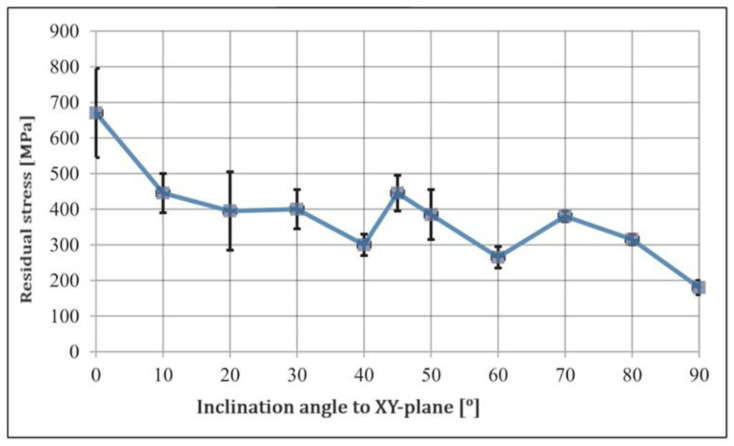
Surface residual stress analyses for SLM-processed Ti-6Al-4V in the as-built condition as a function of the build orientation [70]. (Reused with license number 5643561343784).

Residual stress is the internal stress formed during manufacturing, impacting a component’s structural integrity [88,91,92]. It can lead to premature failure or distortion of the material. Managing residual stress is crucial for ensuring optimal performance and longevity of the part. If these residual stresses exceed the yield strength of a material, it could lead to adverse effects on the service life of the part [91]. Residual stress can be treated by post-process activities, but not fully healed. Defects such as cracking due to residual stress are not mended by post-treatments [55]. Due to the cyclic heating and cooling that occurs during the SLM process, residual stresses are produced [90]. According to Yan et al. [92], utilizing support structures during the SLM process helps to minimize the residual stresses and distortions of the component, preventing unintended component failures. The study by Zyl et al. [93] on residual stress in SLM-built Ti6Al4V reveals a higher residual stress observed in samples without support structures. Moreover, the residual stress in SLM is affected by build direction. The study assessed SLM-processed Ti-6Al-4V in the as-built condition, and the XRD measurement results as a function of build orientation are shown in Figure 4. The condition immediately following SLM processing exhibits high residual tensile stresses, with a peak at a 0° inclination to the x–y axis. By using shot peening during HIP, high compressive stresses were created within the surface layers.

**Figure 5:**
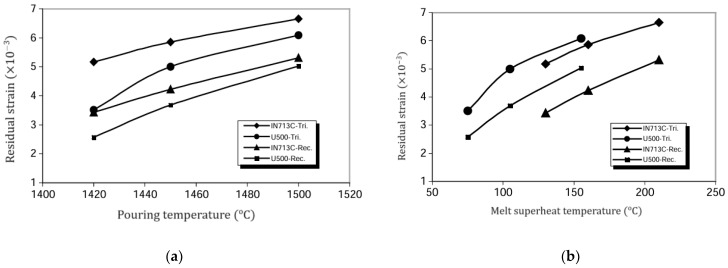
Residual strain as (**a**) a function of pouring temperature, (**b**) melt superheat temperature. (Reused by permission from [94], license number 5643570616839).

The type and number of residual stresses have an impact on casting quality, as it causes undesirable distortions and dimensional changes in vital parts and components. Factors like pouring temperature and geometry also have an effect. The influence of pouring temperature on residual stress in rectangular and triangular shapes of IN713 and U500 produced by investment casting were studied [93]. The result indicates that increasing melted superheat temperature and pouring temperature lead to higher residual stress, as shown in Figure 5a,b. In addition, more residual stress was shown in the triangular samples due to local variation in casting modulus.

**Figure 6:**
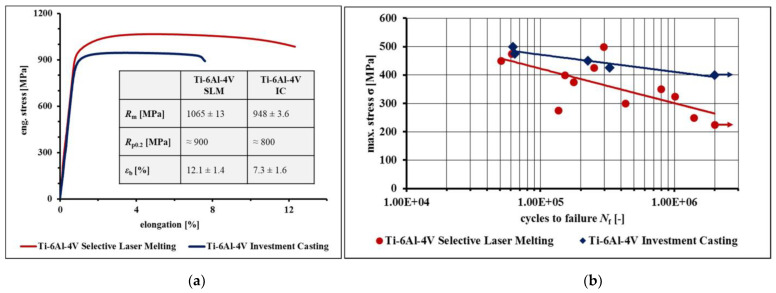
(**a**) Monotonic stress–strain curves for Ti-6Al-4V, processed with selective laser melting and investment casting. (**b**) *S*-*N* curves for Ti-6Al-4V, processed by selective laser melting and investment casting, respectively. The details concerning the different post-treatments are given in the experimental details. (Reused by permission from [70], license number 5643561343784). (**a**,**b**).

According to the comparative investigation by Leuder et al. [70] on SLM- and investment casting-manufactured Ti-6Al-4V lab scale specimens, the monotonic stress–strain performance of SLM parts was superior to investment casting, as shown in Figure 6a. However, as per their findings for loadings in the high-cycle fatigue regime, this contrast was due to flaws caused by the manufacturing process of the SLM parts. Small irregularities in terms of microstructure or geometric notch effects affected the performance of parts under cyclic load, as indicated in Figure 6b. For the fatigue load exposure, the investment casting-manufactured parts were more effective compared to the SLM parts produced with current technology.

## 6. Outlook

The use of SLM technology in manufacturing has led to significant advancements, particularly in the production of complex and intricate parts. However, there are several challenges associated with SLM that limit its ability to entirely replace traditional manufacturing methods. One of the primary challenges is the difficulty in producing large components using SLM. The cost and equipment limitations in this regard make it impractical for certain industries and applications. Currently, SLM technology is better suited for producing smaller and more intricate components. It is expected that large-scale production tools and processes will emerge, enabling the replacement of heavy forgings and castings with 3D-printed components. This potential transformation could revolutionize industries that rely on such bulky and resource-intensive manufacturing methods.

Another significant challenge of SLM technology is its high energy consumption, which begins with the powder preparation stage and continues through material selection and the control of process parameters. Reducing energy consumption and optimizing these stages of the SLM process is a critical area for improvement. While SLM has made inroads into industrial manufacturing and has partially replaced traditional methods in certain applications, it is not without its limitations. There are inherent disadvantages and restrictions associated with the current state of the technology. These limitations include issues related to surface roughness, residual stress within printed parts, and the ability of SLM-produced components to withstand fatigue loads. These aspects require further research and development to enhance the overall quality and performance of SLM-manufactured parts.

Despite these challenges and limitations, the future of SLM appears promising. Ongoing research efforts from various angles and continuous technological improvements are gradually paving the way for SLM to replace traditional casting techniques more effectively. As advancements are made in addressing issues like surface finish, material properties, and energy efficiency, SLM has the potential to become a more competitive option in the manufacturing industry. Ultimately, SLM will likely continue to evolve and expand its role in the manufacturing world, offering a compelling alternative to traditional manufacturing methods.

## 7. Conclusions

This article reviews recent research on comparative study of SLM and investment casting for thin-walled parts applications and research advancements. The review focused on SLM-based AM technology; the following observations are made.

- The tensile strength of SLM-produced parts was relatively better than investment casting-produced parts, while investment casting-produced parts are shown to have better performance under fatigue load. The presence of rough surfaces and voids causes crack initiation, which leads to low fatigue load resistance. SLM has the capacity to replace investment casting, but its performance under cyclic load and ductility needs further investigation. Even though investment casting is an old manufacturing technology, the achievable tight dimensions and the surface qualities of parts produced by this technology are superior to those produced by the current technology SLM under controlled process parameters Surface roughness is one of the challenges and research concerns in SLM, as it forms due to several involved factors.
- The microstructure conditions of SLM and investment casting are highly affected by the involvement of various process parameters. The microstructure of investment casting is relatively coarse due to the slow melt solidification at casting temperature, while SLM produces a fine dendritic structure due to rapid solidification. Microstructure condition has another implication on the difference in tensile strength.
- One of the typical flaws in SLM components is porosity, which has a significant impact on their performance. Porosity increases the susceptibility to corrosion, such as crevice corrosion and pitting corrosion. Even though theoretically SLM can produce structures with up to 100% nominal densities, this is highly reliant on setting the operating conditions, such as the laser power, scan spacing, scan rate, and layer thickness, correctly. Porosity is a well-known drawback of investment casting, which is associated with casting shrinkage, but it is still a serious problem. The formation of porosity in both SLM and investment casting is a very common and serious challenge. However, a comparative study of those manufacturing techniques based on their level of porosity formation does not receive coverage.
- Due to the cyclic heating and cooling that occurs during the SLM process, residual stresses are produced. A supporting structure during the SLM process helps to minimize the residual stress and distortion of parts. In the case of investment casting, factors like pouring temperature and geometry have an effect on the formation of residual stress. However, under the same treatments, SLM parts are shown to have more residual stress.
- Rapid investment casting (RIC) overcomes the labor-intensive and lead-time aspects of traditional casting techniques while producing complex metal components with very good precision. With this connection, 3D printing delivers supreme design flexibility while significantly reducing lead times and prices. For industries looking for quick, accurate, and affordable solutions for complex metal parts, rapid investment casting is a crucial tool. However, RIC faces challenges, including surface roughness and material limitations, which require ongoing research and development to fully employ its capabilities and address these limitations.

## Figures and Tables

**Table 1:** The mechanical properties of SLM and investment casting-produced parts from some previous studies. AlSi7Mg0.6 alloy (reused by permission from [73], license number 5643131487779).

Properties	SLM Y-Axis	SLM Z-Axis	Investment Casting
Yield strength (MPa)	314 ± 79	282 ± 19	271 ± 2
UTS (MPa)	446 ± 6.0	435 ± 18	321 ± 2
E (GPa)	62 ± 4.0	62 ± 2.0	65 ± 1
Elongation (%)	6.1 ± 0.3	3.0 ± 1.0	2.9 ± 0.3
CoCrMoFe [80]			
Rp 0.2%	731.50 ± 40.31		276.20 ± 43.60
Rupture stress (MPa)	1127.91 ± 0.15		391.03 ± 88.91
Max. Stress (MPa)	1136.95 ± 0.92		453.62 ± 75.91
Elongation (%)	13.73 ± 5.32		8.37 ± 4.45
E (GPa)	276.69 ± 12.63		291.21 ± 15.22
Micro Hardness (HV)	420.62 ± 21.16		365.74 ± 16.15
Gold jewellery [77]			
UTS (MPa)	474.2		414.9
Density (g/cm^3^)	15.24		15.26
Elongation (%)	33.5		42.4

## Data Availability

Not available.
